# Family Functioning of Addicted and Non-Addicted Individuals: A Comparative Study

**DOI:** 10.5812/ijhrba.7514

**Published:** 2012-11-26

**Authors:** Mohsen Hosseinbor, Nour-Mohammad Bakhshani, Mansour Shakiba

**Affiliations:** 1 Department of Clinical Psychology and Psychiatry, Zahedan University of Medical Sciences, Zahedan, IR Iran; 2 Research Center for Children and Adolescents Health, Department of Clinical Psychology and Psychiatry, Zahedan University of Medical Sciences, Zahedan, IR Iran

**Keywords:** Family, Substance Abuse, FAD

## Abstract

**Background:**

Family functioning is considered to have a significant impact on the beginnings and maintenance of substance use.

**Objectives:**

The main purpose of this study was to examine and compare the dimensions of family functioning among addicted and non-addicted individuals.

**Patients and Methods:**

In this cross-sectional study, the study sample consisted of 228 individuals, including 118 addicted and 110 non-addicted subjects. The addicted persons were recruited from patients who attended the Baharan Psychiatric Outpatient Clinic for treatment of substance dependence disorders and 110 non-addicted (normal) individuals who were selected from normal populations (students, staff of the hospital and people accompanying patients without a history of substance use) through convenience sampling. The Family Assessment Device (FAD) was used to assess six dimensions of family functioning. The data were analyzed using descriptive indexes (ie, frequency, mean and standard deviation) and t test.

**Results:**

The results showed significant differences in the mean scores of family functioning dimensions including; problem solving, communication, roles, affective responsiveness, affective involvement, behavioral control and overall family performance (P < 0.01).

**Conclusions:**

Results of this study confirmed the lower functioning levels of substance dependent clients’ families on all subscales of the FAD, than in families of non-addicted individuals. It is therefore concluded, that providing interventional strategies for the prevention and treatment of substance use that focus on and involve families in the delivery of health care services is a necessity.

## 1. Background

The definition of family as the fundamental entity in society has been adopted by most researchers and theoreticians (1). Family can provide the basic grounds for the initiation and promotion of both health and illnesses in individuals. Numerous studies have revealed its role and influence on the formation of the concepts of health and illness and on the presentation of models for normal and abnormal behavior (2). Consequently, the family is known as the main and first entity for health (3). According to Bandura, humans are social creature and their behavior should be pondered in light of their social relationships, and it is the family in which one first begins to socialize. Based on dominant viewpoints of family psychology, the psyche and personality are formed within the family, and so it can be considered as the place where learning is first experienced. Moreover, it is also where the majority of anxieties, failures, successes and even aberrations arise, as family members exchange a range of behavioral, affective, and emotional models with each other and negotiate their circumstances, symptoms and sensibilities (4). In order to explain the etiology and continuance of drug abuse, family systems theory emphasizes parents’ drug abuse behavior, weak parental skills, and lack of appropriate organization. A study has shown the fundamental role of numerous variants related to family behavior and functioning, in the prevention and modeling of drug abuse disorders (5). Numerous mechanisms have been suggested in order to explain the reasons for the development of drug abuse behaviors in adolescents. Adolescents may acquire drug abuse behaviors through direct modeling. In addition, adolescents whose parents use drugs are more likely to have a positive attitude towards this issue. Accordingly, this can lead to drug abuse in the case of availability. Moreover, drug abuse behavior by one of the parents and the reaction of the other to such a difficult and chronic problem will probably weaken their power to create an appropriate and organized environment within the family, thus increasing the risk of drug abuse in their children. Another theory presented is the G-dependency theory, which claims that parents who use drugs themselves, support drug use (6). Freidman et al (7) studied 2750 addicts and stated that a direct and reciprocal relationship can be observed between problems existing among family members and addiction problems in the children. Family, as the first influential source during childhood and adolescence, plays a decisive role in the process of making decisions to indulge in high-risk behaviors. During recent years, several studies have been done on the role played by the family in drug abuse, some of which have put more emphasis on the potential influence of family structure on drug abuse and the commitment of other aberrations. A few of the above-mentioned studies, have investigated the functions of the family. In their long-term research, Shedler and Block found that 34% of participants had had no experience of using drugs, 42% had tried them and 24% were dependent on drugs (8). This study showed that the people who became dependent on drugs, had suffered severe affective problems in their developmental stages and the main index of their environment was proposed to be the parents’ low competence in expressing their feelings appropriately. In these families, the children were criticized continuously and were expected to act beyond their actual strength and ability levels. Eitle conducted research concerning; family structure, the level of drug use and relationships, among drug-user adolescents of the same age on 51263 individuals and concluded that weak parental control, and low attachment between parents and children makes addiction and drug use predictable (9). Some theories, explain drug abuse according to parental styles, they believe that the absence of clear and injunctive rules on drug abuse, low parental control, and the lack of suitable punishment following the violation of rules, may at first result in drug use and then later in permanent drug abuse and dependency (10). Dissociation in the family, the quality of parent-child relationships, and parental supports, feedbacks and restrictions on using drugs are known and influential factors in drug abuse (11, 12). In general, abnormal family functioning is considered to be an influential factor in the development and maintenance of drug use. Thus this study was designed and conducted to compare the family functioning of addicted persons and normal ones.

## 2. Objectives

The main purpose of this study was to examine and compare dimensions of family functioning among addicted and non-addicted individuals.

## 3. Patients and Methods

This was a cross-sectional study, conducted during 2011 in Zahedan, Iran. The study sample included 228 participants, 118 of whom had a substance dependence disorder according to the Diagnostic and Statistical Manual of Mental Disorders (DSM), but without a personality disorder or psychotic disorder. Subjects were recruited from patients who attended Baharan Psychiatric Center for the treatment of substance dependence disorders, during November and December 2011. The controls were 110 non-addicted subjects without a history of substance use or psychiatric disorders, and they were selected from normal populations (students, staff of the hospital and people accompanying patients without substance use). In order to assess different family functions, we used the Family Assessment Device (FAD). The FAD is a valid and reliable questionnaire designed by Epstein, Baldwin, and Bishop, it is based on a qualitative study according to the Mc Master Model. The FAD has adequate internal reliability and validity, and its Chronbach’s alphas on the subscales range from 0.74 to 0.92 (13). This assessment contains 60 questions and it is intended to assess family functioning. Six dimensions of family performance including; problem-solving, relationships, roles, affective responsiveness, affective involvement, and behavioral control are specified. The FAD, therefore, consists of six subscales to be assessed, and furthermore, the seventh subscale is related to the overall performance of the family. Every question in the test is provided with options from 1: ‘I totally agree’, to 4: ‘I totally disagree,’ and the questions on descriptions of malfunction are graded vice versa (5). The data were analyzed by using SPSS version 17 software.

## 4. Results

A total of 228 individuals were involved in this study; 118 (51.75%) of whom were drug dependent and 110 (48.25%) non-dependent, 152 (66.7%) were male and 76 (33.3%) were female, A total of 48% of the individuals were single and 52% were married. From an educational point of view, the majority had a high school education (approximately 54%) and approximately 6% had a primary education. *Tables 1 and 2*, illustrate the subjects’ demographical characteristics including: gender, age, education and marital status, along with the frequency and standard deviation of the participants’ dependent and non-dependent variables.

**Table 1:** Frequency of Participants by Gender and Age in the Substance Dependent Group

	No. (%)	Minimum Age, y	Maximum Age, y	Mean ± SD
**Substance Dependent Group**				
**Gender**		16	55	29.43 ± 7.81
Male	97 (82.2)			
Female	21 (17.8)			
**Non-Dependent (Normal) Group**				
**Gender**		17	50	27.99 ± 8.54
Male	55 (50)			
Female	55 (50)			

**Table 2:** Frequency of Participants by Marital Status and Education

	Dependent, No. (%)	Non-Dependent, No. (%)
**Education**		
Primary	7 (5.9)	4 (3.6)
Junior high	40 (33.9)	15 (13.6)
Senior high	56 (47.5)	66 (60)
Academic	15 (12.7)	25 (22.7)
**Marital status**		
Single	56 (47.5)	54 (49.1)
Married	62 (52.5)	56 (50.9)

The minimum and maximum age in the group of drug dependent individuals was 16 and 55 years and the average was approximately 29 years (*Table 1*). In the group of non-dependent individuals, on the other hand, the minimum and maximum of age were reported to be 17 and 50 years, respectively, and the average was approximately 27 years (*Table 1*). Comparison of the average performance of families in the dependent and non-dependent groups showed that dependent individuals scored higher levels on the subscales of family performances and the differences between the two groups were significant (P < 0.01). *Table 3* provides the t-test results.

**Table 3:** The t-Test Results, Means and Standard Deviations of Substance Dependent and Non-Dependent Samples on FAD Scales

	Mean ± SD	*t*	*P*
**Problem solving**	** **	4.97	0.000
Dependent	15.34 ± 3.56	*** ***	*** ***
Non-dependent	13.33 ± 2.37	*** ***	*** ***
**Communication**		3.89	0.000
Dependent	15.65 ± 3.46	*** ***	*** ***
Non-dependent	14.09 ± 2.47	*** ***	*** ***
**Role**		6.13	0.000
Dependent	21.25 ± 3.97	*** ***	*** ***
Non-dependent	18.62 ± 2.17	*** ***	*** ***
**Affective responsiveness**		4.70	0.000
Dependent	15.89 ± 3.72	*** ***	*** ***
Non-dependent	13.83 ± 2.79	*** ***	*** ***
**Affective involvement**		5.99	0.000
Dependent	19.11 ± 4.72	*** ***	*** ***
Non-dependent	15.91 ± 3.09	*** ***	*** ***
**Behavioral control**		5.78	0.000
Dependent	21.90 ± 3.05	*** ***	*** ***
Non-dependent	19.79 ± 2.40	*** ***	*** ***
**Overall performance**		5.18	0.000
Dependent	29.88 ± 7.04	*** ***	*** ***
Non-dependent	25.62 ± 5.14	*** ***	*** ***

Therefore, drug users on most subscales of family performance; problem solving, communication, role, affective responsiveness, affective involvement, behavioral control and overall performance, were different from non-users. A comparison of the subscales of family performance in the dependent and non-dependent groups, on the basis of gender, showed no significant differences in any of the subscales of FAD between men and women, and this indicates that they had the same attitude toward the performances of their families, (*Table 4* and *Table 5*).

**Table 4:** Results of t - Test, Means and Standard Deviations of Substance Dependent Male and Female Samples on FAD Scales

	Mean ± SD	*t*	*P*
**Problem solving**	** **	1.40	0.1
Male	15.13 ± 3.62	*** ***	*** ***
Female	16.33 ± 3.15	*** ***	*** ***
**Communication**		-0.50	0.6
Male	15.57 ± 3.47	*** ***	*** ***
Female	16.00 ± 3.46	*** ***	*** ***
**Role**		1.23	0.2
Male	21.46 ± 3.91	*** ***	*** ***
Female	20.28 ± 4.17	*** ***	*** ***
**Affective responsiveness**		0.24	0.8
Male	15.93 ± 3.72	*** ***	*** ***
Female	15.71 ± 3.77	*** ***	*** ***
**Affective involvement**		0.02	0.9
Male	19.11 ± 4.57	*** ***	*** ***
Female	19.09 ± 4.48	*** ***	*** ***
**Behavioral control**		-0.62	0.5
Male	21.82 ± 3.03	*** ***	*** ***
Female	22.28 ± 3.19	*** ***	*** ***
**Overall performance**		0.36	0.7
Male	30.00 ± 7.11	*** ***	*** ***
Female	29.38 ± 6.80	*** ***	*** ***

**Table 5:** Results of t-Test, Means and Standard Deviations of Non-clinical (Normal) Male and Female Samples on FAD Scales

	Mean ± SD	*t*	*P*
**Problem solving**	** **	0.6	0.5
Male	13.47 ± 2.12	*** ***	*** ***
Female	13.20 ± 2.62	*** ***	*** ***
**Communication**	14.47 ± 2.37	1.62	0.1
Male		*** ***	*** ***
Female		*** ***	*** ***
**Role**		-1.54	0.1
Male	18.30 ± 2.49		
Female	18.94 ± 1.77		
**Affective responsiveness**		1.16	0.2
Male	14.14 ± 2.86		
Female	13.52 ± 2.71		
**Affective involvement**		0.83	0.4
Male	16.16 ± 3.37		
Female	15.67 ± 2.80		
**Behavioral control**		0.83	0.4
Male	19.98 ± 2.56	*** ***	*** ***
Female	19.60 ± 2.24	*** ***	*** ***
**Overall performance**		1.05	0.2
Male	26.14 ± 5.21	*** ***	*** ***
Female	25.10 ± 5.07	*** ***	*** ***

## 5. Discussion

The results of this study, indicate that dependent individuals, compared to non-dependent ones, report more negative ideas about their family’s performance, which is in accordance with previous findings (5, 14-17). According to these results, the families of dependent individuals have lost their efficiency in areas such as; problem solving, interactions and communications (15, 17, 18), affective responsiveness and involvement (3, 5, 15, 19, 20), behavioral control (16, 19) and they lack the competence to find appropriate solutions to problems in a way that does not distress individual members (14, 17). The inefficiency of these families is considerably more likely to lead to drug abuse and this is in accordance with the findings of Sajida and Hamid (21) and Logan, Walker et al. (22). Inefficient families function as obstacles to the development and mental maturity of their members, and this is due to; disturbed affective relationships, lack of competence to cope with problems and the ability to find appropriate and logical solutions to them, failing to be supportive, and not fulfilling physical and affective needs, which causes them to become more and more susceptible to using drugs (23). Additionally, according to the findings of Jesoor (24), Eitel (9), Springer et al. (23), the core of the problematic behaviors and neurotic disorders lies in connection and its quality, combined with healthy relationships in the family and those between parents and their children. Individuals who do not have an intimate and supportive relationship with their family are more likely to be attracted by and inclined towards friends and groups of their own age and become more susceptible to using drugs (5, 12, 18, 23). Other studies on the relationship between family performance and running away from home (25, 26), psychiatric problems (14, 26-29) and sexual harassment have shown that, in families with impaired performance, drug abuse is more frequent. Feeling insecure, caused by a loss in the efficiency of the family, creates a direct and significant relationship with using drugs. In order to explain these findings, it is worth mentioning that in families where negative relationships exist and parents who do not involve themselves in their children’s lives and problems, the development of a strong self-concept and the reinforcement of competence and self-control in children are hindered, moreover they seek to be connected with their peers more and more; all of which makes them more inclined to use drugs. Newcomb and Richardson (12) found that intimate, friendly and supportive relationships reduce an adolescent’s inclination to use drugs. Based on the psychoanalytic viewpoint by Kohut (30), parents, and especially the mother’s, inattention to the needs of the child causes them to fail to regulate their behaviors and stresses, to remain dependent on surrounding circumstances and matters, and therefore, they are vulnerable to using drugs due to their inner-mental deficiencies. No significant difference was found in the explanations and functioning of families between men and women, which indicates that they share the same assessment on the performance of their families. Some limitations of the current research must be considered in the generalizability of these results. First of all, no casual inferences can be derived from this research, because we used a cross-sectional method. As a result, we suggest future longitudinal studies to examine the impact of family functioning on the development of substance abuse. In addition, the FAD is a self-report scale which must take into account the problems of using self-report questionnaires when interpreting the results.
